# Subjective Happiness Scale: Measurement properties of the online and paper-pen administrations in Nepali adults with musculoskeletal pain

**DOI:** 10.1016/j.bjpt.2025.101245

**Published:** 2025-08-08

**Authors:** Ritu Basnet, Anupa Pathak, Mark P. Jensen, Narendra Singh Thagunna, James H. McAuley, Saurab Sharma

**Affiliations:** a Department of Psychology, K and K International College, Tribhuvan University, Nepal; b Department of Physiotherapy, Scheer Memorial Adventist Hospital, Banepa, Nepal; c Department of Psychiatry and Mental Health, Institute of Medicine, Tribhuvan University Teaching Hospital, Kathmandu, Nepal; d School of Public Health, Faculty of Medicine and Health, University of Sydney, Camperdown, Sydney, NSW, Australia; e Department of Rehabilitation Medicine, University of Washington, Seattle, WA, USA; f Department of Psychology, Padma Kanya Multiple Campus, Tribhuvan University, Nepal; g Pain Management and Research Centre, Royal North Shore Hospital, Northern Sydney Local Health District, Sydney, NSW, Australia; h Pain Management Research Institute, Kolling Institute, Faculty of Medicine and Health, The University of Sydney and Northern Sydney Local Health District, Sydney, NSW, Australia; i School of Health Sciences, Faculty of Medicine and Health, University of New South Wales, Sydney, Australia; j Centre for Pain IMPACT, Neuroscience Research Australia, Sydney, Australia

**Keywords:** Happiness, musculoskeletal pain, low back pain, Developing countries, Reliability, Validity

## Abstract

**Background:**

Happiness is a positive psychological construct often described as subjective well-being. It is associated with a meaningful life, and better social support and coping with stress or trauma. Happiness may have a role in buffering the negative effects of musculoskeletal pain on quality of life. Validating measures that assess subjective happiness in individuals with musculoskeletal pain can help advance research and patient care in this emerging field.

**Objective:**

We sought to: (1) evaluate the measurement properties of the Subjective Happiness Scale (SHS) in a sample of Nepali adults with musculoskeletal pain; and (2) compare its measurement properties when administered using hard-copy and online methods.

**Methods:**

The Consensus-based Standards for the selection of health status Measurement Instruments (COSMIN) guidelines informed the conduct and reporting. A total of 180 (120 hard-copy and 60 online administrations) individuals with musculoskeletal pain were recruited in Nepal. Content validity, structural validity (exploratory factor analysis), internal consistency (Cronbach’s alpha), construct validity (hypothesis testing), and test-retest reliability (Intraclass Correlation Coefficient, ICC_2,1_), measurement error were assessed.

**Results:**

Single factor structure of the SHS was supported. The SHS showed good internal consistency for the combined, hard-copy, and online samples (Cronbach’s alphas 0.857, 0.848, and 0.847, respectively). It evidenced moderate to good test-retest reliability [ICCs = 0.86 (95 % CI: 0.80, 0.93), 0.89 (95 % CI: 0.82, 0.93), and 0.66 (95 % CI: 0.32, 0.87), respectively]. The findings also supported the construct validity for both administration types.

**Conclusions:**

This study supports the validity of the SHS for assessing subjective happiness in adults with musculoskeletal pain, with moderate to good reliability.

## Highlights

• Happiness may be an important protective factor in musculoskeletal pain.
• Nepali Subjective Happiness Scale (SHS-NP) has one factor solution.
• Item #4 was incomprehensible by Nepali adults.
• SHS-NP is reliable and valid tool to assess subjective happiness.
• Responsiveness and minimum important change needs further testing.

## Introduction

Musculoskeletal pain contributes more to global disability than any other condition.^1^, ^2^, ^3^, ^4^ Musculoskeletal pain is a prevalent condition accounting for significant cause of disability in many low- and middle-income countries, including Nepal.^3^, ^4^, ^5^ It is influenced by a myriad of biopsychosocial and cultural factors.^6^, ^7^, ^8^, ^9^, ^10^, ^11^, ^12^, ^13^ Historically, the focus of psychological pain research has been on “negative” or maladaptive psychological factors. There has been a recent interest in examining the possible benefits of positive psychological factors, which focus on the individual’s strength and qualities of personal growth and flourishing in buffering the negative effects of pain.^14^^,^^15^

Happiness is one such positive psychological construct. The United Nations consider happiness as a fundamental human goal.^16^ Happiness is self-reported and is commonly termed as subjective happiness. Merriam-Webster Dictionary defines happiness as a state of well-being and contentment. It is a complex concept, often described as and used interchangeably with subjective well-being, and is a component of overall quality of life.^17^^,^^18^ It is commonly associated with meaningful life, better social support, and better coping with stress and trauma.^19^ It has both affective (infrequent instances of negative affect and frequent instances of positive affect) and cognitive (high level of satisfaction in life) components.^20^ Evidence indicates that subjective happiness can affect subjective pain sensitivity and is associated with activity in brain that underlies the affective component of pain.^21^^,^^22^

The Subjective Happiness Scale (SHS) was developed in 1999 in high school students and community adults using reflexive model.^17^ It is a commonly used instrument for assessing happiness. As of 28^th^ November 2024, the primary paper describing its development had over 6398 citations on Google Scholar.^17^ The original version of the SHS is a 4-item scale with items to assess global subjective happiness. It has been translated into more than 15 languages, including Arabic, Bangla, Brazilian Portuguese, Chilean, Chinese, French, German, Greek, Hungarian, Italian, Japanese, Lebanese, Mexican, Serbian, Spanish, and Turkish.^23^, ^24^, ^25^, ^26^, ^27^, ^28^, ^29^, ^30^, ^31^, ^32^, ^33^, ^34^, ^35^, ^36^, ^37^, ^38^, ^39^, ^40^, ^41^ The SHS has been shown to have inconsistent factor structure, good to excellent internal consistency and test-retest reliability, and good construct validity as indicated by moderate to strong associations with measures of subjective well-being, resilience, self-efficacy, health-related quality of life, depression, and physical health.^24^^,^^30^^,^^32^^,^^41^ Although several scales assessing happiness have been developed since the development of the SHS, they lack robust measure development procedures, are much longer, and do not have superior measurement properties to SHS.^42^, ^43^, ^44^, ^45^

To evaluate the potential role of happiness as a construct that could contribute to positive adjustment in individuals with musculoskeletal pain, a reliable and valid measure of happiness (e.g., the SHS) in the population of interest (i.e. musculoskeletal pain in this case), is a pre-requisite.^46^ Although the SHS has been shown to be valid in both healthy samples (e.g., general populations, students, and working women^25^^,^^40^^,^^47^) and samples of individuals with a variety of health conditions (e.g., chronic kidney diseases^48^ and patients with depressive disorders^41^), its measurement properties have not yet been evaluated in a sample of Nepali adults with musculoskeletal pain. Given these considerations, the aim of this study was to assess the measurement properties of SHS in a sample of Nepali adults with musculoskeletal pain in Nepal. Specifically, we sought to evaluate its content validity, internal consistency, test-retest reliability, measurement error, and construct validity. Further, given the increasing use of online administration of measures in pain research, a secondary aim was to determine if an online administration of SHS would evidence similar reliability and validity as a hard-copy version.

## Methods

The study protocol was pre-registered in Open Science Framework (https://osf.io/k3gbe/). We conducted this study in two phases. We first translated and cross-culturally adapted the original English language version of the SHS into Nepali using state-of-the-science translation guidelines.^49^ Next, we evaluated the measurement properties of the Nepali version of SHS in a sample of Nepali adults with musculoskeletal pain using Consensus-based Standards for the selection of health status Measurement Instruments (COSMIN) recommendations.^46^^,^^50^, ^51^, ^52^ The Institutional Review Committee (IRC) of Scheer Memorial Adventist Hospital in Nepal (Reference number: 39/21) approved the protocol. Written consent was obtained from each participant who could read and write before collecting data. For participants who were not literate, verbal consent was obtained, and a witness signed on their behalf. The study sample also provided data to evaluate the measurement properties of the Pain Self-Efficacy Questionnaire the results of which are reported elsewhere.^53^

### Phase 1: translation and cross-cultural adaptation

**Fig. 1:**
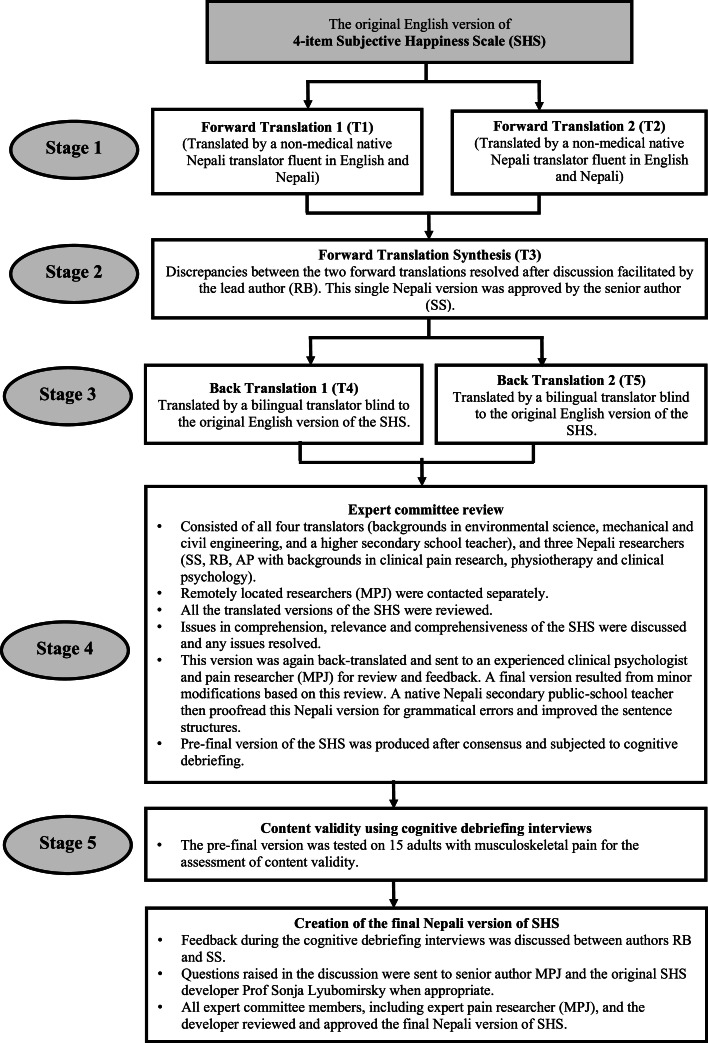
Translation procedures.

We formally translated SHS into Nepali using recommended guidelines by Beaton and colleagues and Functional Assessment of Chronic Illness Therapy (FACIT) methodology.^49^^,^^54^ The translation process we used is summarized in Fig. 1.

#### Content validity

The first author (RB) conducted cognitive interviews on 15 adults with musculoskeletal pain using a standardized interview guide and data collection forms previously adapted from FACIT and Patient-Reported Outcomes Measurement Information System (PROMIS) methodologies (see **Supplementary Material - S1**).^54^^,^^64^ SS, highly experienced in cognitive interviews for evaluating patient-reported outcome measures,^55^, ^56^, ^57^, ^58^ trained RB. The aims of the interviews were to evaluate the comprehensibility, comprehensiveness, relevance, and cultural appropriateness of the instructions, items, and response options and identify any problematic items, and areas to improve readability of text. Participants were encouraged to think aloud to better understand their comprehension of the scale.^54^ All cognitive interviews were audio-recorded and key components of the interviews relevant to study questions were transcribed verbatim. The interviewer also took fieldnotes as needed. COSMIN guidelines consider a sample size of seven or more participants adequate for pre-testing and cognitive interviews.^59^^,^^60^

Comprehensiveness and relevance were further discussed among the expert committee team members (including the study authors) who have clinical psychology and physical therapy backgrounds.

### Phase 2: evaluation of measurement properties

#### Study design and setting

We used an observational longitudinal test-retest design to evaluate the measurement properties of the SHS in the study sample. Data were collected using two methods: a hard-copy pencil-and-paper method (*n* = 120) and an online survey (*n* = 60). Participants who completed the hard-copy version of questionnaire were recruited from the outpatient department of Scheer Memorial Adventist (*n* = 85) and the community of Bagmati Province, Nepal (*n* = 35); participants who completed the online versions (*n* = 60) were recruited from one of the author’s (RB) clinical practices who volunteered to complete online questionnaires.

#### Participants

To participate in the current study, potential participants needed to be 18 years or older and have had musculoskeletal pain in any location (e.g., neck, shoulder, back, knee) with an average pain intensity in the past week of at least 3 out of 10 on a 11-point Numerical Pain Rating Scale where 0 = “No pain” and 10 = “Maximum pain”.^61^ Individuals who could not understand Nepali or had a recent surgery that could have been the primary cause of their current pain (i.e., surgery within the past three months) were excluded.^62^ COSMIN guidelines recommend at least 100 participants for assessment of measurement properties.^52^^,^^63^ For the analysis of factor structure, the recommended sample size requirement for the participants-to-variables ratio is 10:1.

#### Measures

The participants were also asked to complete Nepali versions of 10 additional measures assessing nine validity criteria (see Table 1).

**Table 1:** Description of the study measures.

Domain	Measures in Nepali	Description of the measure	Cronbach’s alpha in the current study
Subjective Happiness	The Subjective Happiness Scale	The Subjective Happiness Scale The original SHS has four items and assesses global happiness. ^17^ Respondents are asked to rate each item on 1 to 7 Likert scales, with 1 the lowest level of happiness and 7 representing a high level of happiness. A sample item is “In general, I consider myself: 1 = “Not a very happy person”, 7 = “A very happy person”. The responses for all items are summed into a total score, which can range from of 4 to 28, with higher scores indicating greater happiness.^17^	–
Depressive symptom severity	PROMIS® Depression Short Form 8b Scale	Participants were asked to rate the frequency with which they experienced each depressive symptom in the past seven days using a five-point scale with 1 = “Never,” 2 = “Rarely,” 3 = “Somewhat,” 4 = “Often,” and 5 = “Always.” Higher scores indicate a greater frequency of depressive symptoms. The Nepali version of the scale has shown to be reliable and valid.^56^^,^^64^	0.92
Pain intensity	PROMIS® Pain Intensity Short Form 3a Scale	The responses were recorded on a 5-point Likert scale where 1= “No pain” and 5= “Very severe pain,” with higher scores indicating greater pain intensity. The Nepali version of the scale has shown to be reliable and valid.^56^^,^^64^	0.78
Pain interference	PROMIS® Pain Interference Short Form 6b Scale	Participants were asked to rate the interference of pain in each listed activity using a 1 to 5 Likert scale, where 1= “Not at all,” 2 = “A little bit,” 3 = “Somewhat,” 4 = “Quite a bit,” and 5 = “Very much.” Higher scores indicate greater pain interference. The Nepali version of the scale has shown to be reliable and valid.^56^^,^^64^	0.92
Sleep disturbance	PROMIS® Sleep Disturbance Short Form 8b Scale	The participant's responses for the first four items were rated as 1 = “Not at all,” 2 = “A little bit,” 3 = “Somewhat,” 4 = “Quite a bit,” and 5 = “Very much.” The responses for the next three items are rated as 1 = “Never,” 2 = “Rarely,” 3 = “Sometimes,” 4 = “Often,” and 5 = “Always.” The last item is rated using response options 1 = “Very poor,” 2 = “Poor,” 3 = “Fair,” 4 = “Good,” and 5 = “Very good.” Higher scores indicate greater sleep disturbance. The Nepali version of the scale has shown to be reliable and valid^56^^,^^64^	0.91
Pain-related catastrophizing	Pain Catastrophizing Scale	The PCS has three subscales assessing pain-related rumination, magnification, and helplessness on a 5-point scale ranging from 0 (“Not at all”) to 4 (“Always”). Higher scores indicate more frequent catastrophizing. The Nepali PCS has shown to be reliable and valid.^58^	0.93
Physical function	Patient-Specific Functional Scale	Participants first identified three activities most important to them and rate their ability to engage in these activities now, relative to their ability to engage in the activity before the onset of the pain problem, on a numerical scale from 0 (“Unable to perform the activity”) to 10 (“Able to perform the activity at the same level as before injury or problem”). Higher scores indicate less impact of the pain problem on the respondent’s three favorite activities. The Nepali PSFS has shown to be reliable, valid, and responsive.^55^	0.80
Pain self-efficacy	10-item Pain Self-Efficacy Questionnaire	Participants were asked to respond to each item using a 7-point Likert scale from 0 ("Not at all confident") to 6 ("Completely confident"). Higher scores indicate greater pain self-efficacy. The Nepali PSEQ has shown to be reliable and valid.^53^	0.95
Resilience	2-item Connor Davidson Resilience Scale	Participants indicate the extent to which each resilience item is true for them on a 5-point Likert scale ranging from 0 (“Not true at all”) to 4 (“True nearly all the time”). Higher scores indicate more resilience. The Nepali version of the scale has shown to be reliable and valid.^65^	0.56
Perceived global quality of life	2-item quality of life scale	Participants were asked to rate their global perceived quality of life using 2 items "*In general, how would you rate your overall quality of life during the past week?"* and "*How would you rate your general health during the past week?"* Participants indicate their agreement with these statements using a 5-point ordinal scale, where greater scores indicate a better perceived overall quality of life.^66^^,^^67^	0.65
Perceived global rating of change scale	Global Rating of Change Scale (GROC)	GROC asked the question “Thinking about how you had described your pain during the first assessment, how does the pain feel now?”. It was scored on a seven-point Likert scale, with a mid-point score of 4 representing “No change,” scores less than 4 indicate worsening pain and scores greater than 4 indicate improved pain.^61^ Here, responses to the measure were used to classify the participants into those who rated improvement (i.e., responses ≥5) from those who reported that their pain was stable (i.e., GROC score = 4) for assessing reliability and estimating measurement errors. As pain is the main complaint in people with musculoskeletal pain, stability of pain was considered. This measure has been used previously to classify participants as stable and improved groups.^55^^,^^58^^,^^61^^,^^64^^,^^68^^,^^69^	N/A

***Abbreviations*:** PROMIS®; SEM, Standard Error of Measurement; SDC, Smallest Detectable Change.

***Note:*** All the measures demonstrated adequate reliability and validity.

#### Study procedures

Participants were asked to complete questionnaires at an initial assessment and again at a follow-up assessment with the Global Rating of Change (GROC) at an interval of 3 to 14 days using either hard-copy or online methods. We considered three days adequate to avoid recall bias, especially when approximately 60 items were administered (excluding pain and demographic questions).^70^^,^^71^ Assistance was provided to the hard-copy respondents when needed. For all participants who completed the hard-copy version of the questionnaires at the initial assessment, the second assessment was completed either in-person interview or, for those who could not visit the hospital, by phone call, to reduce attrition. Phone assessments are commonly used in research, and we have previously successfully used phone calls to obtain follow-up data in prior studies.^61^^,^^68^^,^^69^ All the participants who completed the questionnaire online at the baseline assessment also completed the questionnaire online at follow-up.

The participants who indicated that they preferred to complete an online version of the questionnaires were sent the Google Form link via the participant's preferred medium (e.g., email). The Google Form included the screening questions for eligibility, informed consent, demographic information, and the study questionnaires. The follow-up assessment for online administration was done by sending the pre-filled Google Form link with demographic information using the same online platform. Participants who opted to be reminded for follow-up assessment were given reminders (one to five times) via telephone, email, or social media for the online follow-up assessment.

### Data analyses

#### Content validity

The audio recordings, transcripts, and field notes were reviewed by two independent authors (RB with AP or SS). The data analyses were guided by interpretative content analysis principles to ensure comprehensibility and cultural appropriateness of instructions, items, responses, and comprehensiveness and relevance of items to musculoskeletal pain conditions.^60^ Selective coding of the transcripts was performed during the analysis to achieve the interview aims as needed.^60^

#### Measurement properties

We first computed descriptive statistics (means and standard deviations of continuous variables and numbers and percentages of categorical variables) for the entire sample and separately for the sub-samples to describe the sample and study measures. We then compared the sub-samples using *t*-tests or Mann-Whitney U Test for continuous variables depending on data normality, or chi-squared tests for categorical variables.

*Factor analysis.* We then conducted exploratory factor analysis to evaluate the factor structure of SHS using Principal Axis Factoring as factor structure of the 3-item SHS we evaluated (due to exclusion of one of the original items, see below) is currently not known. The Kaiser-Meyer-Olkin measure of sampling adequacy and Bartlett's test of sphericity were computed to examine the appropriateness of data for factor analysis. We considered a Kaiser-Meyer-Olkin value of greater than 0.60 and item loadings with value greater than 0.50 to indicate that the data were acceptable for factor analysis. Components with eigenvalues > 1 were retained and plotted on a Cattell's Scree plot.

*Reliability.* To evaluate the reliability of the SHS, we computed Cronbach’s alphas for the entire sample and the sub-samples. We considered internal consistencies from 0.60 to 0.69 as marginal, 0.70 to 0.79 as adequate, 0.80–0.89 as good, and 0.90 or more as excellent.^72^ We then computed Intraclass Correlation Coefficients (ICC) using the two-way random effect model and absolute agreement to evaluate the test-retest reliability of the SHS, again for both the entire sample and for the sub-samples, for participants within the stable group only (i.e. GROC = 4). We considered the ICC value less than 0.50 as poor, 0.50 to 0.74 as moderate, 0.75 to 0.89 as good, and 0.90 or more as excellent.^73^

*Measurement error.* Measurement error was assessed in participants who were stable (GROC = 4). We calculated the standard error of measurement (SEM_agreement_) of the SHS using the formula SEM = SD_change_ x √(1 - ICC) ^74^ for the entire sample and the two sub-samples. We computed smallest detectable change (SDC_agreement_) using the formula 1.96 x √2 x SEM.^74^

*Construct validity.* We evaluated construct validity using hypothesis testing approach recommended by COSMIN guidelines^46^^,^^50^ by computing nine Pearson Correlation Coefficients (based on the results of normality tests presented in **Supplementary Material - S2**) between the baseline SHS and the nine validity criterion scores. All hypotheses were finalized before pre-registration and any analyses were conducted. A pattern of moderate to strong negative associations of SHS were expected with measures of depressive symptom severity and positive associations with measures of resilience and perceived global quality of life.^27^^,^^30^^,^^35^^,^^41^ As no previous research existed for generating hypotheses for remaining measures, research team determined that at least weak negative associations of SHS were expected with the measures of pain intensity, pain interference, sleep disturbance, pain catastrophizing, and positive association with measure of physical function and pain self-efficacy. We planned to conclude that the SHS evidenced construct validity if at least 75 % of the hypotheses (that is seven of nine hypotheses) were supported, as per the recommendation of the COSMIN guidelines.^46^^,^^50^ We considered a correlation coefficient of < 0.30 as indicating a weak association, 0.30 to 0.49 as indicating a moderate association, and values of ≥ 0.50 as indicating a strong association.^75^

## Results

### Phase 1: content validity

Fifteen participants with diversity in age, education level, birth sex, and ethnic identities agreed to participate in cognitive debriefing interviews. One participant was excluded because of a lack of overall comprehension. The median years of education was 12 (interquartile range 4.5 – 16). Three participants (21 %) never went to school. Additional demographic characteristics of the cognitive debriefing sample are presented in **Supplementary Material - S2.**

The participants confirmed the comprehensiveness of the scale as no new concepts relevant to the concept of subjective happiness were suggested. There were no changes needed for the relevance of the scale instruction, items, and responses. Two of the SHS items (Items 3 and 4) required cross-cultural adaptation. Thirteen out of 14 participants (93 %) failed to understand the meaning of SHS item 4, due in large part because it is a reverse scored item; it asks participants to indicate the extent to which a description of someone who is not very happy describes them. As a result, this item was removed before subjecting it to the measurement testing phase after discussion with the scale developer. Details of cross-cultural adaptation process are reported in **Supplementary Material – S3** and the final Nepali SHS is included as Appendix 1.

### Phase 2: measurement properties

#### Demographic and pain characteristics

The mean age of the combined sample was 40.8 (*SD*, 15.1) years. The majority of participants were women (69 %), married (75 %), Hindu (86 %), and financially independent (77 %). The median years of education was 13 (interquartile range, 9 – 17) where 25 participants (14 %) never went to school.

Almost half of the participants (46 %) reported that they had pain in multiple sites, and the low back was the most commonly reported site (*n* = 104, 58 %), followed by lower extremity (*n* = 83, 46 %) and upper extremity (*n* = 48, 27 %). The median pain duration was 1 year (Range=0.01 to 30 years, interquartile range = 0.16 – 4.0). Thirty-seven percent of the participants in the combined sample reported pain daily.

The mean age was significantly lower, and the median years of education was significantly higher for online sample compared to the hard-copy sample. Significant differences between the hard-copy and online samples were also observed for occupation, degree of financial independence, pain difficulty frequency, and comorbidities (all p’s<0.05). Additional details of demographic and pain characteristics of the total sample and sub-samples are presented in Table 2. The average recall period for follow up assessment was 8.6 (standard deviation 2.4) days.

**Table 2:** Demographic variables of the participants in the measurement properties testing samples.

Variable	Frequency ( %) or Mean (SD) or Median (IQR)			*p*-value
	Total	Pen-and-paper	Online	
Number recruited	179 (100 %)	119 (67 %)	60 (34 %)	
Follow-up assessment interval (days)	8.59 (2.41)	8.47 (2.47)	8.85 (2.47)	0.34
Sex identified at birth				
Female	123 (69 %)	84 (70 %)	39 (65 %)	0.45
Male	56 (31 %)	35 (30 %)	21 (35 %)	
Age in years	40.84 (15.13)	44.42 (15.82)	33.75 (10.60)	< 0.001
Marital Status				
Married	134 (75 %)	94 (79 %)	40 (67 %)	0.07
Single	45 (25 %)	25 (21 %)	20 (33 %)	
Religion				
Hindu	154 (86 %)	104 (87 %)	50 (84 %)	0.57
Buddhist	18 (10 %)	10 (9 %)	8 (13 %)	
Christian	7 (3.4 %)	5 (4 %)	2 (3 %)	
Self-reported ethnicity				
Chhetri	79 (44 %)	55 (46 %)	24 (40 %)	0.45
Newar	55 (31 %)	34 (29 %)	21 (35 %)	
Brahmin	32 (18 %)	20 (17 %)	12 (20 %)	
Tamang	11 (6 %)	9 (7 %)	2 (3 %)	
Others	2 (1 %)	1 (1 %)	1 (2 %)	
Years of Education*	13 (9 – 17)	12 (5 – 16)	16 (13 –17.75)	<0.001
No school	25 (14 %)	25 (20 %)	0	
Primary school	13 (7 %)	12 (11 %)	1 (2 %)	
Secondary school	23 (13 %)	19 (16 %)	4 (7 %)	
Higher secondary school	17 (9.5 %)	11 (9 %)	6 (10 %)	
Bachelor’s degree (13–15)	53 (29.5 %)	25 (21 %)	28 (47 %)	
Master’s degree and above (>15)	48 (27 %)	27 (22 %)	21 (35 %)	
Occupation				
Office worker	49 (27 %)	25 (21 %)	24 (40 %)	0.003
Nurse	33 (18 %)	19 (16 %)	14 (23 %)	
Homemaker	25 (14 %)	22 (18 %)	3 (5 %)	
Unemployed	13 (7 %)	10 (8 %)	3 (5 %)	
Agriculture worker	12 (7 %)	12 (10 %)	0	
Retired	11 (6 %)	9 (8 %)	2 (3 %)	
Student	9 (5 %)	3 (2 %)	6 (10 %)	
Other	27 (16 %)	19 (17 %)	8 (14 %)	
Financial Independence				
Yes	137 (77 %)	85 (71 %)	52 (87 %)	0.023
No	42 (23 %)	34 (29 %)	8 (13 %)	
Family Income per month⁎⁎				
No income	2 (1 %)	1 (1 %)	1 (2 %)	0.27
Less than NRs. 10,000	5 (3 %)	4 (3 %)	1 (2 %)	
Between NRs. 10,001 - 30,000	33 (19 %)	25 (21 %)	8 (13 %)	
Between NRs. 30,001 - 50,000	56 (31 %)	32 (27 %)	24 (40 %)	
Between NRs. 50,001 - 100,000	38 (21 %)	23 (19 %)	15 (25 %)	
More than NRs. 100,001	45 (25 %)	35 (29 %)	11 (18 %)	
Self-defined family income category				
Not enough to provide family	9 (5 %)	5 (4 %)	4 (7 %)	0.77
Enough to provide family	50 (28 %)	34 (29 %)	16 (27 %)	
Save a little in a month	91 (51 %)	59 (49 %)	32 (53 %)	
Save half of the income in a month	29 (16 %)	21 (18 %)	8 (13 %)	
Pain Site				
Single site	96 (54 %)	67 (56 %)	29 (48 %)	0.31
Multiple sites	84 (46 %)	52 (44 %)	31 (52 %)	
Low back	104	75	29	
Lower extremity	83	52	31	
Upper extremity	48	29	19	
Upper back	20	16	4	
Neck	14	9	10	
Pain duration in years*	1.0 (0.16 – 4.0)	1.49 (0.49 – 4.0)	0.5 (0.04 – 3.0)	0.007
Pain difficulty frequency				
Everyday	67 (37 %)	37 (31 %)	30 (50 %)	0.038
Half the time since pain onset	66 (37 %)	50 (42 %)	16 (27 %)	
Less than the time since pain onset	46 (26 %)	32 (27 %)	14 (23 %)	
Pain due to injury or accident				
No	167 (93 %)	111 (93 %)	56 (93 %)	0.99
Yes	12 (7 %)	8 (7 %)	4 (7 %)	
Co-morbidities				
No	108 (60 %)	60 (50 %)	48 (80 %)	<0.001
Yes	71 (40 %)	59 (50 %)	12 (20 %)	
Hypertension	25	21	4	
Diabetes	15	14	1	
Osteoarthritis	18	16	2	
Arthritis	8	4	4	
Other	29	21	8	
Treatment				
Yes	109 (61 %)	74 (62 %)	35 (58 %)	0.62
Medication	68	55	13	
Physiotherapy	84	64	20	
Home treatment	30	15	15	
Other	1	0	1	
No	70 (39 %)	45 (38 %)	25 (42 %)	
SHS score (out of 21)				
Initial assessment	15.96 (3.18)	15.51 (3.15)	16.85 (3.08)	0.008
Follow-up assessment	16.31 (3.14)	16.10 (2.94)	16.77 (3.53)	0.195

**Abbreviations:** IQR, Interquartile Range; SD, Standard Deviation; SHS, Subjective Happiness Scale.

**Note:** Multiple responses are allowed in Pain sites, Co-morbidities, and Any Treatment.

⁎ Data were not normally distributed and therefore are presented as Median and IQR.

⁎⁎ 1 United States Dollar = 132.24 Nepalese Rupees (16/2/2023).

#### SHS scores

One of 180 enrolled participants who provided data via hard-copy administration did not provide any responses to the SHS items, leaving 179 participants (119 in the hard-copy sub-sample and 60 in the online subsample) for all analyses. The means and SDs of SHS from the initial assessments and the follow-up assessments for the combined samples were 15.96 (3.18) and 16.31 (3.14), respectively. Additional means and SDs of SHS for sub-samples are presented in Table 2.

#### Exploratory factor analysis

Bartlett’s Test of Sphericity was found to have a significant correlation (*p* = <0.01) among its components. The results of the EFA obtained a single-factor solution for the 3-item SHS. The factor loadings were >0.70 for all three items in all three sample groups. Details about the factor loadings are presented in **Supplementary Material – S4**.

#### Reliability and measurement error

The results of the reliability and measurement error analyses are presented in Table 3. The internal consistency was good for combined, hard-copy, and online samples (Cronbach's alpha range from 0.847 to 0.857). The results of test-retest reliability analyses (ICC_2,1_) in the stable group for the combined and hard-copy samples were good, while ICC_2,1_ for online sample was marginal. Similarly, measurement error parameters were greater for the online sample compared to the total and pen-and-paper methods.

**Table 3:** Reliability and measurement error of the 3-item SHS.

SHS (Score out of 21)	Sample (N)	Stable group (N)	Internal consistency Cronbach’s alpha	*Test-retest reliability (ICC)	SD for mean change score	*SEM	*SDC_95 %_
Total	179	72	0.857	0.857 (0.804, 0.929)	1.98	0.75/21	2.08/21
Pen-and-paper	119	60	0.848	0.892 (0.820, 0.931)	1.70	0.55/21	1.52/21
Online	60	12	0.847	0.661 (0.322, 0.873)	2.88	1.68/21	4.65/21

**Abbreviations:** SHS, Subjective Happiness Scale; ICC, Intraclass correlation coefficient; SEM, Standard Error of Measurement; SD, Standard Deviation; SDC, Smallest Detectable Change.

Note:.

⁎ Test-retest reliability and measurement error were conducted in the stable sample only with global rating of change score of 4.

#### Validity

The construct validity of the 3-item SHS in the combined and two sub-samples was confirmed as 89 % (8 out of 9) of *a priori* hypotheses were supported for the combined sample and online samples, while 78 % (7 out of 9) of the *a priori* hypotheses were supported for the hard-copy sample (see Table 4).

**Table 4:** Correlation coefficients (*r*) between the Nepali version of 3-item SHS validity variables.

Scales	*A priori* hypothesis	Total sample	Hypotheses met?	Hard-copy	Hypotheses met?	Online sample	Hypotheses met?
**PROMIS® Depression Symptom Severity**	Negative moderate to strong association	−0.40⁎⁎	Yes	−0.37⁎⁎	Yes	−0.46⁎⁎	Yes
**PROMIS® Pain Intensity**	At least weak negative association	−0.12	No	−0.04	No	−0.23	Yes
**PROMIS® Pain Interference**	At least weak negative association	−0.26⁎⁎	Yes	−0.24⁎⁎	Yes	−0.31*	Yes
**PROMIS® Sleep Disturbance**	At least weak negative association	−0.29⁎⁎	Yes	−0.29⁎⁎	Yes	−0.32*	Yes
**PCS**	At least weak negative association	−0.22⁎⁎	Yes	−0.20*	Yes	−0.24	Yes
**PSFS**	At least weak positive association	0.21⁎⁎	Yes	0.14	No	0.19	No
**PSEQ**	At least weak positive association	0.45⁎⁎	Yes	0.49⁎⁎	Yes	0.40⁎⁎	Yes
**CD-RISC-2**	Positive moderate to strong association	0.40⁎⁎	Yes	0.36⁎⁎	Yes	0.54⁎⁎	Yes
**2-item Quality of Life Scale**	Negative moderate to strong association	0.44⁎⁎	Yes	0.46⁎⁎	Yes	0.38⁎⁎	Yes
**Total number of hypotheses met.** **n ( %)**			8 (89 %)		7 (78 %)		8 (89 %)

**Abbreviations:** SHS, Subjective Happiness Scale; PROMIS®, Patient-Reported Outcomes Measurement Information System; PCS, Pain Catastrophizing Scale; PSFS, Patient Specific Functional Scale; CD-RISC-2, 2-item Connor Davidson Resilience Scale; PSEQ, Pain Self-Efficacy Questionnaire.

⁎ *p* < 0.05.

⁎⁎ *p* < 0.01.

## Discussion

This study aimed to assess the measurement properties of the SHS in individuals with musculoskeletal pain. We found that the reverse-coded fourth SHS item was not comprehensible by the vast majority of the cognitive debriefing participants and was therefore removed from the final version, with the approval of the scale developer. The findings support the reliability and validity of the 3-item SHS for assessing perceived happiness in a sample of individuals with musculoskeletal pain via hard-copy and online administrations.

### Implications

The findings have important research and clinical implications. First, the findings support the use of SHS in research studying subjective happiness in individuals with musculoskeletal pain as it is reliable and valid self-reported measure to assess subjective happiness. Second, the findings suggest that more reliable assessment could be obtained by administering a hard-copy version of the SHS compared to online administration—although the findings do indicate that online administration would still provide valid results. Third, the findings may help facilitate a global understanding of the role of subjective happiness in pain management when conducting between-country and cross-cultural research and contribute to development of interventions to effectively treat musculoskeletal pain.

### Comparison with previous studies

Incomprehension related to the reverse wording of item #4 and its low factor loading is common,^29^^,^^35^^,^^36^^,^^39^^,^^76^ with the resulting 3-item scale demonstrating acceptable reliability and validity.^41^^,^^77^ Our findings related to one-factor solution of SHS is consistent with published research.^24^, ^25^, ^35^ The internal consistency and test-retest reliability coefficients are also comparable to those found in studies of English-speaking and non-English speaking samples.^17^^,^^23^^,^^29^^,^^39^ Construct validity for the measure was supported with a pattern of positive associations with measures of resilience and quality of life and negative associations with depression, consistent with prior research.^24^^,^^30^^,^^31^^,^^35^

### Comparisons between the hard-copy and online administrations

The findings indicated that online administration resulted in similar validity as the hard-copy administration, but lower reliability and greater measurement error. The reasons for the differences could potentially be because of (1) the differences between the study samples (different sources of samples with significantly different demographic profiles); or (2) the participants in the online administration condition were not offered assistance to complete the measures. Although in-person administration is recommended, online administration of SHS could still obtain valid results.

### Strengths

We followed the standard guidelines for conducting rigorous translation, cross-cultural adaptation, measurement properties evaluation, and study reporting using COSMIN guidelines.^50^ We also recruited adequate sample sizes with minimal loss to follow-up (6 %). To our knowledge, this is the first study to (1) assess the measurement properties of SHS in individuals with chronic pain, (2) compare the measurement properties between hard-copy and online administrations, and (3) assess the measurement error of the SHS.

### Limitations

First, cognitive debriefing testing was conducted face-to-face only and not online. As a result, we do not know whether online participants would provide any different feedback. Second, when determining that sub-sample that was “stable” for estimating test-retest reliability, we classified participants based on the GROC scale responses related to no change in *pain*—the main clinical complaint in the target population with musculoskeletal pain, and not change in *subjective happiness*, which would have been ideal. Given the follow-up maximum duration of two weeks, we considered the duration inadequate to change the construct of happiness. Future research may use a GROC scale assessing perceived change in happiness for selecting the participants for the test-retest ability analysis, if possible, and build on to the evidence-base of reliability of SHS, which would likely result in a larger reliability estimate than found in this study.

Third, we did not evaluate the responsiveness of SHS in people who report changes in overall happiness when no intervening treatment is provided. Future research is needed to (1) evaluate the responsiveness and minimum important change of SHS to natural changes in happiness over time, (2) determine if the SHS is sensitive to treatment-related changes in subjective happiness, and (3) determine if such changes mediate the beneficial effects of pain treatments on other outcomes.

Fourth, to minimize attrition, we administered the second SHS using interviews conducted over the telephone (only 6 % of total participants), which differed from the initial data collection method. Such an approach has been successfully used in other studies of measurement properties^53^^,^^69^^,^^78^, ^79^, ^80^ and does not affect measurement properties of the scale in question. Fifth, the number of participants via online recruitment were smaller than in person recruitment (*n* = 60 vs. 119). Larger sample sizes are recommended for online recruitment to ensure robust measurement properties for online administration.

Sixth, the findings may not necessarily generalize to populations (e.g., children) or with other health conditions or individuals who live in other countries. Additional research is also needed to evaluate the measurement properties of SHS in other pain populations, including but not limited to pediatric populations, populations with other pain conditions, and populations who live in other countries and who speak other languages, in order to understand the overall generalizability of findings. Finally, although we provided preliminary evidence on content validity through interviews of the target population, additional research should further explore this by interviewing health professionals and experts in happiness and well-being.

## Conclusions

The findings support a one factor structure for the 3-item SHS evaluated here. Our study findings support the reliability and content and construct validity of the 3-item SHS for assessing subjective happiness in individuals with musculoskeletal pain. Internal consistency is good for both hard-copy and online administration (i.e., Cronbach’s alphas ≥ 0.84). Although test-retest reliability is marginal for online administration of the measure, it is good for hard-copy administration (ICC = 0.89). Overall, the study findings provide preliminary support that the 3-item SHS can provide reliable and valid results for use when testing the role of happiness in adjusting to musculoskeletal pain.

## Funding

This research did not receive any specific grant from funding agencies in the public, commercial, or not-for-profit sectors. SS was supported by the International Association for the Study of Pain John J. Bonica Postdoctoral Fellowship during the time of manuscript writing.

## Declaration of competing interest

The authors declare no competing interest.

## Appendix B Supplementary materials

Image, application 1

## References

[1] Ferreira M.L., de Luca K., Haile L.M. (2023). Global, regional, and national burden of low back pain, 1990–2020, its attributable risk factors, and projections to 2050: a systematic analysis of the Global Burden of Disease Study 2021. Lancet Rheumatol.

[2] Steinmetz J.D., Culbreth G.T., Haile L.M. (2023). Global, regional, and national burden of osteoarthritis, 1990–2020 and projections to 2050: a systematic analysis for the Global Burden of Disease Study 2021. Lancet Rheumatol.

[3] Gill T.K., Mittinty M.M., March L.M. (2023). Global, regional, and national burden of other musculoskeletal disorders, 1990–2020, and projections to 2050: a systematic analysis of the Global Burden of Disease Study 2021. Lancet Rheumatol.

[4] Sharma S., McAuley J.H. (May 2022). Low back pain in Low- and middle-income countries, part 1: the problem. J Orthop Sports Phys Ther.

[5] Sharma S., Jensen M.P., Pathak A., Sharma S., Pokharel M., Abbott J.H. (Nov-Dec 2019). State of clinical pain research in Nepal: a systematic scoping review. Pain Rep.

[6] Dunn M., Rushton A.B., Soundy A., Heneghan N.R. (2022). Individuals’ beliefs about the biopsychosocial factors that contribute to their chronic musculoskeletal pain: protocol for a qualitative study in the UK. BMJ open.

[7] Gatchel R.J., Peng Y.B., Peters M.L., Fuchs P.N., Turk D.C. (Jul 2007). The biopsychosocial approach to chronic pain: scientific advances and future directions. Psychol Bull.

[8] Turk D.C., Wilson H., Swanson K.S., Ebert M., Kerns R. (2011). The biopsychosocial model of pain and pain management. Behav psychopharmacol pain manag.

[9] Nicholas M.K. (Nov 1 2022). The biopsychosocial model of pain 40 years on: time for a reappraisal?. Pain.

[10] Reis F.J.J., Nijs J., Parker R., Sharma S., Wideman T.H. (Sep-Oct 2022). Culture and musculoskeletal pain: strategies, challenges, and future directions to develop culturally sensitive physical therapy care. Braz J Phys Ther.

[11] Sharma S., Abbott J.H., Jensen M.P. (2018). Why clinicians should consider the role of culture in chronic pain. Braz J Phys Ther.

[12] Sharma S., Ferreira-Valente A., Williams A.C., Abbott J.H., Pais-Ribeiro J., Jensen M.P. The role of culture in pain-related beliefs, coping, and catastrophizing: a systematic review. PROSPERO 2017 CRD42017082449.

[13] Sharma S., Pathak A., Jensen M.P. (2016). Words that describe chronic musculoskeletal pain: implications for assessing pain quality across cultures. J Pain Res.

[14] Bartley E.J., Makhoul M., Palit S., Robinson M.E., Fillingim R.B. (May 2, 2023). Examining physical and cognitive function in chronic low back pain through the use of a multisystem resilience framework. Pain Med.

[15] Schroeter A.C., MacDonald D.A., Scholten-Peeters G.G.M., Goubert L., Kendall E., Coppieters M.W. (Oct 29 2022). Preferred self-administered questionnaires to assess resilience, optimism, pain acceptance, and social support in people with pain: a modified Delphi study. Pain Med.

[16] Helliwell J.F., Layard R., Sachs J.D., De neve J.E., Aknin L.B., Wang S. (2024).

[17] Lyubomirsky S., Lepper H.S. (1999). A measure of subjective happiness: preliminary reliability and construct validation. Soc Indic Res.

[18] Land K.C., Michalos A.C., Sirgy M.J. (2011).

[19] Lyubomirsky S., King L., Diener E. (Nov 2005). The benefits of frequent positive affect: does happiness lead to success?. Psychol Bull.

[20] Diener E., Suh E.M., Lucas R.E., Smith H.L. (1999). Subjective well-being: three decades of progress. Psychol Bull.

[21] Yoshino A., Okamoto Y., Onoda K. (Apr 15 2010). Sadness enhances the experience of pain via neural activation in the anterior cingulate cortex and amygdala: an fMRI study. Neuroimage.

[22] Zhao H., Chen A.C. (2009). Both happy and sad melodies modulate tonic human heat pain. J Pain.

[23] Dogan T., Totan T. (2013). Psychometric properties of Turkish version of the Subjective Happiness Scale. J Happiness Well-Being.

[24] Nan H., Ni M.Y., Lee P.H. (Aug 2014). Psychometric evaluation of the Chinese version of the Subjective Happiness Scale: evidence from the Hong Kong FAMILY Cohort. Int J Behav Med.

[25] Quezada L., Landero R., González M.T. (2016). A validity and reliability study of the Subjective Happiness Scale in Mexico. J Happiness Well-Being.

[26] Iani L., Lauriola M., Layous K., Sirigatti S. (2014). Happiness in Italy: translation, factorial structure and norming of the subjective happiness scale in a large community sample. Soc Indic Res.

[27] Swami V. (2008). Translation and validation of the malay subjective happiness scale. Soc Indic Res.

[28] Shimai S., Otake K., Utsuki N., Ikemi A., Lyubomirsky S. (Oct 2004). [Development of a Japanese version of the Subjective Happiness Scale (SHS), and examination of its validity and reliability]. Nihon Koshu Eisei Zasshi.

[29] Szabo A. (2019). Validity of the Hungarian version of the subjective Happiness Scale (SHS-HU). Mentálhig és Pszichoszomatika.

[30] Karakasidou E., Pezirkianidis C., Stalikas A., Galanakis M. (2016). Standardization of the subjective happiness scale (SHS) in a greek sample. Psychology.

[31] Kotsou I., Leys C. (2017). Echelle de bonheur subjectif (SHS): propriétés psychométriques de la version française de l’échelle (SHS-F) et ses relations avec le bien-être psychologique, l’affect et la dépression. Can J Behav Sci/Rev can sci du comport.

[32] Jovanović V. (2014). Psychometric evaluation of a Serbian version of the Subjective Happiness Scale. Soc Indic Res.

[33] Moghnie L., Kazarian S.S. (2012). Subjective happiness of Lebanese college youth in Lebanon: factorial structure and invariance of the Arabic Subjective Happiness Scale. Soc Indic Res.

[34] Vera-Villarroel P., Celis-Atenas K., Cordova-Rubio N. (2011). Evaluation of happiness: psychometric analysis of the subjective happiness scale in Chilean population. Ter Psicol.

[35] Extremera N., Fernández-Berrocal P. (2014). The Subjective Happiness Scale: translation and preliminary psychometric evaluation of a spanish version. Soc Indic Res.

[36] Chien C.-L., Chen P.-L., Chu P.-J., Wu H.-Y., Chen Y.-C., Hsu S.-C. (2020). The Chinese version of the Subjective Happiness Scale: validation and convergence with multidimensional measures. J Psychoeduc Assess.

[37] Swami V., Stieger S., Voracek M., Dressler S.G., Eisma L., Furnham A. (2009). Psychometric evaluation of the Tagalog and German subjective happiness scales and a cross-cultural comparison. Soc Indic Res.

[38] Islam R.M., Ahmed O., Naher L., Akter M. (2020). The subjective Happiness scale: a psychometric evaluation in Bangladesh context. Bangladesh Psychol Stud.

[39] Ortiz F.R., Paiva S.M., Pordeus I.A., Ardenghi T.M. (Apr 22 2021). Psychometric properties and longitudinal measurement invariance of the Brazilian version of the subjective happiness scale in adolescents. J Clin Transl Res.

[40] Alquwez N., Cruz J.P., Alotaibi N.S., Alshammari F. (2021). Validity and reliability of the subjective happiness scale arabic version among Saudi working women. J Taibah Univ Med Sci.

[41] Feliu-Soler A., de Diego-Adelino J., Luciano J.V. (Oct 19 2021). Unhappy while depressed: examining the dimensionality, reliability and validity of the subjective happiness scale in a Spanish sample of patients with depressive disorders. Int J Env Res Public Health.

[42] Hills P., Argyle M. (2002). The Oxford Happiness Questionnaire: a compact scale for the measurement of psychological well-being. Pers Individ Dif.

[43] Salas-Vallina A., López-Cabrales Á., Alegre J., Fernández R. (2017). On the road to happiness at work (HAW) transformational leadership and organizational learning capability as drivers of HAW in a healthcare context. Pers Rev.

[44] Sanli E., Celik S.B., Gencoglu C. (2019). Validity reliab authentic happiness scale.

[45] Rizzato M., Di Dio C., Miraglia L. (Aug 19 2022). Are you happy? A validation study of a tool measuring happiness. Behav Sci (Basel).

[46] Mokkink L.B., Elsman E.B.M., Terwee C.B. (2024). COSMIN guideline for systematic reviews of patient-reported outcome measures version 2.0. Qual Life Res.

[47] Almaleki D.A. (2021). The psychometric properties of distance-digital subjective happiness scale. Int J Comput Sci Netw Secur.

[48] Sousa L.M.M., Vieira C.M.A.M., Severino S.S.P., Pozo-Rosado J.L., José H.M.G. (2017). Validation of the subjective happiness scale in people with chronic kidney disease. Enferm Glob.

[49] Beaton D.E., Bombardier C., Guillemin F., Ferraz M.B. (2000). Guidelines for the process of cross-cultural adaptation of self-report measures. Spine (Phila Pa 1976).

[50] Mokkink L.B., Terwee C.B., Patrick D.L. (May 2010). The COSMIN checklist for assessing the methodological quality of studies on measurement properties of health status measurement instruments: an international Delphi study. Qual Life Res.

[51] Mokkink L.B., Terwee C.B., Gibbons E. (Sep 22 2010). Inter-rater agreement and reliability of the COSMIN (COnsensus-based Standards for the selection of health status Measurement Instruments) checklist. BMC Med Res Methodol.

[52] Mokkink L.B., Terwee C.B., Knol D.L. (Mar 18 2010). The COSMIN checklist for evaluating the methodological quality of studies on measurement properties: a clarification of its content. BMC Med Res Methodol.

[53] Basnet R., Jensen M.P., Pathak A. (Apr 2024). Self-efficacy in Nepali adults with musculoskeletal pain: measurement properties of hard-copy and online versions of the pain Self-efficacy questionnaire. J Pain.

[54] Eremenco S.L., Cella D., Arnold B.J. (Jun 2005). A comprehensive method for the translation and cross-cultural validation of health status questionnaires. Eval Health Prof.

[55] Sharma S., Palanchoke J., Abbott J.H. (Aug 2018). Cross-cultural adaptation and validation of the Nepali translation of the patient-specific functional scale. J Orthop Sports Phys Ther.

[56] Sharma S., Correia H., Pathak A. (Apr 2021). Translation and cross-cultural adaptation of Nepali versions of the patient-reported outcomes measurement information system (PROMIS(R)) pain intensity, pain interference, pain behavior, depression, and sleep disturbance short forms in chronic musculoskeletal pain. Qual Life Res.

[57] Sharma S., Higgins C., Cameron P. (Mar 2022). Validation of the Nepali version of the self-reported Leeds assessment of neuropathic symptoms and signs (S-LANSS) in adults with chronic pain and predominantly low-literacy levels. J Pain.

[58] Sharma S., Thibault P., Abbott J.H., Jensen M.P. (2018). Clinimetric properties of the Nepali version of the Pain Catastrophizing Scale in individuals with chronic pain. J Pain Res.

[59] Wild D., Grove A., Martin M. (Mar-Apr 2005). Principles of good practice for the translation and cultural adaptation process for patient-reported outcomes (PRO) measures: report of the ISPOR Task Force for Translation and Cultural Adaptation. Value Health.

[60] Terwee C.B., Prinsen C.A.C., Chiarotto A. (May 2018). COSMIN methodology for evaluating the content validity of patient-reported outcome measures: a Delphi study. Qual Life Res.

[61] Sharma S., Palanchoke J., Reed D., Haxby Abbott J. (Dec 4 2017). Translation, cross-cultural adaptation and psychometric properties of the Nepali versions of numerical pain rating scale and global rating of change. Health Qual Life Outcomes.

[62] Sharma S., Pathak A., Jha J., Jensen M.P. (2018). Socioeconomic factors, psychological factors, and function in adults with chronic musculoskeletal pain from rural Nepal. J Pain Res.

[63] Prinsen C.A.C., Mokkink L.B., Bouter L.M. (May 2018). COSMIN guideline for systematic reviews of patient-reported outcome measures. Qual Life Res.

[64] Sharma S., Pathak A., Maharjan R., Abbott J.H., Correia H., Jensen M. (2018). Psychometric properties of nepali versions of PROMIS short from measures of pain intensity, pain interference, pain behaviour, depressions, and sleep disturbance. J Pain.

[65] Sharma S., Pathak A., Abbott J.H., Jensen M.P. (Apr 3 2018). Measurement properties of the Nepali version of the Connor Davidson resilience scales in individuals with chronic pain. Health Qual Life Outcomes.

[66] Sharma S., Jensen M.P., Moseley G.L., Abbott J.H. (Aug 10 2018). Pain education for patients with non-specific low back pain in Nepal: protocol of a feasibility randomised clinical trial (PEN-LBP Trial). BMJ Open.

[67] Sharma S., Jensen M.P., Moseley G.L., Abbott J.H. (Mar 27 2019). Results of a feasibility randomised clinical trial on pain education for low back pain in Nepal: the Pain Education in Nepal-low Back pain (PEN-LBP) feasibility trial. BMJ Open.

[68] Kc S., Sharma S., Ginn K., Almadi T., Subedi H., Reed D. (Mar 21 2019). Cross-cultural adaptation and measurement properties of the Nepali version of the DASH (disability of arm, shoulder and hand) in patients with shoulder pain. Journal article. Health Qual Life Outcomes.

[69] Sharma S., Jha J., Pathak A., Neblett R. (Jul 27 2020). Translation, cross-cultural adaptation, and measurement properties of the nepali version of the central sensitization inventory (CSI). BMC Neurol.

[70] Maharjan R., Bovonsunthonchai S., Vachalathiti R. (2024). The STarT back screening tool: the Nepali translation, cross-cultural adaptation and measurement properties in adults with non-specific low back pain. Musculoskelet Care.

[71] Bier J.D., Ostelo R., van Hooff M.L., Koes B.W., Verhagen A.P. (May 1, 2017). Validity and reproducibility of the STarT back tool (Dutch Version) in patients with low back pain in primary care settings. Phys Ther.

[72] Cicchetti D.V. (1994). Guidelines, criteria, and rules of thumb for evaluating normed and standardized assessment instruments in psychology. Psychol Assess.

[73] Koo T.K., Li M.Y. (Jun 2016). A guideline of selecting and reporting intraclass correlation coefficients for reliability research. J Chiropr Med.

[74] de Vet H.C.W., Terwee C.B., Mokkink L.B., Knol D.L. (2011).

[75] Cohen J. (1992).

[76] Zager Kocjan G., Jose P.E., Socan G., Avsec A. (Jun 2022). Measurement invariance of the subjective happiness scale across countries, gender, age, and time. Assessment.

[77] O’Connor B.P., Crawford M.R., Holder M.D (2015). An item response theory analysis of the subjective happiness scale. Soc Indic Res.

[78] Kc S., Sharma S., Ginn K., Almadi T., Reed D. (Aug 30 2019). Nepali translation, cross-cultural adaptation and measurement properties of the Shoulder Pain and Disability Index (SPADI). J Orthop Surg Res.

[79] Zeugfang D., Wisetborisut A., Angkurawaranon C. (Jul 19 2018). Translation and validation of the PACIC+ questionnaire: the Thai version. BMC Fam Pr.

[80] Awwad O., AlMuhaissen S., Al-Nashwan A., AbuRuz S. (2022). Translation and validation of the arabic version of the Morisky, Green and Levine (MGL) adherence scale. PLoS One.

