# Supplementary material for: Subjective Happiness Scale: Measurement properties of the online and paper-pen administrations in Nepali adults with musculoskeletal pain
Source: Braz J Phys Ther. 2025 Aug 8;29(5):101245. doi: 10.1016/j.bjpt.2025.101245 (PMC12355072; doi:10.1016/j.bjpt.2025.101245)
Supplement: Supplementary file 1 [file mmc1.pdf]

Supplementary Materia – S1. Cognitive debriefing interview questions.

1.	(Pilot-testing) पाईलट टेस्टइङ साईट:	
2.	Interview date मिति:	(Day) दिन / (Month) महिना / (Year) वर्ष
3.	(Language) तपाईको घरमा बोल्ने भाषा:	
4.	(Sex) लिंग:	☐ (Female) महिला ☐ (Male) पुरुष
5.	(Date of Birth) जन्म वर्ष:	AD
6.	(Country of Birth) जन्मिएको देश:	
7.	(Years of formal education) तपाईले कति वर्ष औपचारिक शिक्षा लिनुभएको छ ?	
8.	(ethnicity) जातियता	☐ बाह्मण ☐ क्षेत्री ☐ नेवार ☐ तामाङ ☐ राई/ लिम्बु<br>☐ सेर्पा/लामा ☐ दलित ☐ अन्य (प्रस्ट्याउनुहोस्) _____
9.	(Religion) धर्म	
10.	(Address) ठेगाना	Urban/Semi-Urban/Rural (सहरी/ अर्धशहरी / ग्रामीण)
11.	Questionnaire completion?	☐ Self-reported by participant<br>☐ Completed by participants with little assistance<br>☐ Completed by researcher on behalf of the participants
12.	Duration of pain	
13.	Pain site	
14.	Pain intensity (0-10) on numerical scale (where 0=no pain, and 10= worst possible pain) in previous 7 days?	

**NOTE TO INTERVIEWER: Please ask the participant about items that have been previously marked in the questionnaire as difficult to understand. Do your best to work with the participant to make sure that all problematic items are listed. Please ask why the items were difficult to understand or why the participant thought they were difficult.*

Basnet, R., Pathak, A., Jensen, M. P., Thagunna, N. S., McAuley, J. H., Sharma, S. Subjective Happiness Scale: Measurement properties of the online and paper pen administrations in Nepali adults with musculoskeletal pain. (2025). *Brazilian Journal of Physical Therapy*.

1. आफ्नो खुसीको स्केलमा बुझ्न गाह्रो कुनै प्रश्न थिए ?
Were there any items on the subjective happiness scale which were difficult to understand?

☐ थिए (थिए भने, प्रश्न नम्बर 2 मा जानुहोस्)
**Yes (if yes, continue to question 2)*

☐ थिएन (थिएन भने, प्रश्न नम्बर 3 मा जानुहोस्)
**No (if no, skip to question 3)*

2. तपाईं मलाई भन्न सक्नु हुन्छ कि कुन प्रश्न गाह्रो थियो र किन ?
र के तपाईं मलाई यसलाई अझै राम्रो ढंगले कसरी सोध्न मिल्छ भन्न सक्नुहुन्छ ?
**Would you please tell me which items were difficult to understand and why they were difficult? Also, could you suggest a better way to phrase these items?*

प्रश्न नम्बर **Item Number*	*टिप्पणीहरू र सुझावहरू* **Comments and suggestions* Nepali	Translated into English

3. के यस प्रश्नावलीमा नचाहिने र आपत्तिजनक प्रश्नहरु कुनै थिए ?
**Were there any items on the questionnaire which were not relevant or were offensive to you?*

☐ थिए (थिए भने, प्रश्न नम्बर 4 मा जानुहोस्)
**Yes (if yes, continue to question 4)*

☐ थिएन (थिएन भने, प्रश्न नम्बर 5 मा जानुहोस्)
**No (if no, skip to question 5)*

4. के तपाईं मलाई भन्न सक्नु हुन्छ कि कुन प्रश्न नचाहिने वा आपत्तिजन थिए? के तपाईं मलाई यसलाई अझै राम्रो ढंगले कसरी सोध्न मिल्छ भन्न सक्नु हुन्छ ?
**Would you please tell me which items were not relevant or were offensive and why? Also, could you suggest a better way to phrase these items?*

प्रश्न नम्बर | *टिप्पणीहरू र सुझावहरू*
**Comments and suggestions*

Basnet, R., Pathak, A., Jensen, M. P., Thagunna, N. S., McAuley, J. H., Sharma, S. Subjective Happiness Scale: Measurement properties of the online and paper pen administrations in Nepali adults with musculoskeletal pain. (2025). *Brazilian Journal of Physical Therapy*.

Item Number	Nepali	Translated into English

5. के तपाईंलाई अरु पनि केही खुसीको प्रश्न उल्लेख गर्नुपर्छ जस्तो लाग्छ ?
**Do you think we should add other questions about happiness to the questionnaire?*

- ☐ लाग्छ (लाग्छ भने, प्रश्न नम्बर 6 मा जानुहोस्)
  **Yes (if yes, continue to question 6)*
- ☐ लाग्दैन (लाग्दैन, भने, प्रश्न नम्बर 7 मा जानुहोस्)
  **No (if no, skip to question 7)*

6. कृपया भन्नुहोस् अरु केही थप्न अथवा परिवर्तन गर्नपर्छ पर्छ होला ?
**Please tell me what you think we should add?*

प्रश्न नम्बर **Item Number*	*टिप्पणीहरू र सुझावहरू* **Comments and suggestions* Nepali	Translated into English

7. अब म तपाईंलाई केही विशेष प्रश्नहरुको बारे सोध्न जादै छु । यसमा कुनै सहि वा गलत जवाफ छैनन् । म केबल तपाईंको विचार मात्र जान्न चाहन्छु । त्यसैले प्रश्नको अर्थ वर्णन गर्न आफ्नै शब्दहरुमा जवाफ दिनु होला । यो प्रश्नावली अङ्रेजीबाट नेपालीमा अनुवाद भएको हो । तपाईंका सल्लाह र सुझवहरुले यस अनुवादमा आएका त्रुटीहरु पहिचान गर्न मद्दत गर्ने छ ।
**Now I would like to ask you about some items in particular. There are no right or wrong answers to these questions. I would simply like to know your opinion, so please use your own words when answering questions about the meaning of words or phrases. This questionnaire was translated from English into Nepali. Your feedback is appreciated and will help identify problems in the translation.*

आफ्नो खुसीको स्केल (Subjective Happiness Scale)

निर्देशन (Instruction):

तलको प्रत्येक वाक्यहरुमा वा प्रश्नहरुमा तपाईलाई वर्णन गर्ने अङ्क पहिचान गरी त्यसमा गोलो चिन्ह लगाउनुहोस् ।

(For each of the following statements and/or questions, please circle the point on the scale that you feel is most appropriate in describing you.)

8. के निर्देशन गाह्रो अथवा नबुझिने छ र केही परिवर्तन चाहिन्छ?
Was instruction difficult to understand and need any changes?

9. कृपया प्रश्न १ हेर्नुहोस्, "सामान्यतया, म आफूलाई यस्तो व्यक्ति सम्झिन्छु:"
Please look at the item 1: In general, I consider myself:

१	२	३	४	५	६	७
धेरै खुसी नहुने						धेरै खुसी हुने

1	2	3	4	5	6	7
Not a very happy person						a very happy person

9a. तपाईंले कुन अङ्क छान्नु भयो र किन ?
Which number/answer do you choose and why?

9b. के उत्तरका बिकल्पहरु बुझ्नलाई सरल र सजिला छन्? छैनन् भने वर्णन गर्नुहोस् ।
Were options simple and easy to understand? If not please explain.

9c. के तपाइलाई दिइएको विकल्पहरु प्रश्न अनुसार मिलेको जस्तो लाग्छ? लाग्दैन भने किन र विकल्पलाई कसरी प्रश्नसंग मिलाउन सकिन्छ?
Do you think the options given align with the question? If not why and how can options be aligned with questions.

10. कृपया प्रश्न २ हेर्नुहोस्, "मेरो धेरै जसो साथीहरुको तुलनामा मलाई लाग्छ कि म:"
Please look at the item 2: Compared to most of my peers, I consider myself:

१	२	३	४	५	६	७
थोरै खुसी छु						धेरै खुसी छु

1	2	3	4	5	6	7
Less happy						More happy

10a. तपाईंले कुन अङ्क छान्नु भयो र किन?
Which number/answer do you choose and why?

10b. के उत्तरका बिकल्पहरु बुझ्नलाई सरल र सजिला छन्? छैनन् भने वर्णन गर्नुहोस् ।
Were options simple and easy to understand? If not please explain.

10c. के तपाइलाई दिइएको विकल्पहरु प्रश्न अनुसार मिलेको जस्तो लाग्छ ? लाग्दैन भने किन र विकल्पलाई कसरी प्रश्नसंग मिलाउन सकिन्छ?
Do you think the options given align with the question? If not why and how can options be aligned with questions.

11. कृपया प्रश्न ३ हेर्नुहोस्, "कोही मानिसहरू सामान्यतया धेरै खुसी हुन्छन् । जीवनमा उनीहरु जस्तोसुकै परिस्थितिमा पनि रमाउदै आफूले गरेको सबै कुराहरुबाट सन्तुष्टि लिन सक्छन् । यसले तपाईको व्यक्तित्वलाई कुन हदसम्म व्याख्या गर्छ?"
Please look at the item 3: Some people are generally very happy. They enjoy life regardless of what is going on, getting the most out of everything. To what extent does this characterization describe you?

१	२	३	४	५	६	७
अलिकति पनि गर्दैन						धेरै नै गर्छ

1	2	3	4	5	6	7
Not at all						a great deal

11a. तपाईंले कुन अङ्क छान्नु भयो र किन ?
Which number/answer do you choose and why?

11b. के उत्तरका बिकल्पहरु बुझ्नलाई सरल र सजिला छन्? छैनन् भने वर्णन गर्नुहोस् ।
Were options simple and easy to understand? If not please explain.

11.c के तपाइलाई दिइएको विकल्पहरु प्रश्न अनुसार मिलेको जस्तो लाग्छ ? लाग्दैन भने किन र विकल्पलाई कसरी प्रश्न संग मिलाउन सकिन्छ?
Do you think the options given align with the question? If not why and how can options be aligned with questions.

12. कृपया प्रश्न ४ हेर्नुहोस्, “कोही मानिसहरू सामान्यतया धेरै खुसी हुदैनन् । उनीहरु निराशै नभएपनि जति खुसी हुन्छन्, त्यति खुसी कहिल्यै देखिदैनन् । यसले तपाईको व्यक्तित्वलाई कुन हदसम्म व्याख्या गर्छ?”
Please look at the item 4: Some people are generally not very happy. Although they are not depressed, they never seem as happy as they might be. To what extend does this characterization describe you?

	१	२	३	४	५	६	७
अलिकति पनि गर्दैन							धेरै नै गर्छ

	1	2	3	4	5	6	7
Not at all							a great deal

12a. तपाईंले कुन अङ्क छान्नु भयो र किन ?
Which number/answer do you choose and why?

12b. के उत्तरका बिकल्पहरु बुझ्नलाई सरल र सजिला छन्? छैनन् भने वर्णन गर्नुहोस् ।
Were options simple and easy to understand? If not please explain.

12c. के तपाइलाई दिइएको विकल्पहरु प्रश्न अनुसार मिलेको जस्तो लाग्छ ? लाग्दैन भने किन र विकल्पलाई कसरी प्रश्न संग मिलाउन सकिन्छ?
Do you think the options given align with the question? If not why and how can options be aligned with questions.

13. तपाईंका अन्य केही आफ्नो खुसीको स्केल सम्बन्धी सुझावहरु छन् ?
**Do you have any other suggestion regarding the questionnaires?*

☐ छैन (छैन भने प्रश्न 14 मा जानुहोस्)
**No (if no, skip to question 14)*

☐ छ (छ भने तल लेख्नुहोस्)

**Yes (if yes, list them below)*

Nepali	Translated to English
______________________________	______________________________
______________________________	______________________________
______________________________	______________________________

14. हामीलाई यस अनुसन्धानमा मद्दत गर्नुभएकोमा धेरै धेरै धन्यवाद । तपाईंको सुझावले आफ्नो खुसीको स्केल बिभिन्न अन्तर राष्ट्रिय अनुसन्धानमा र बिरामी जाँच्न प्रयोग गर्न सकिने छ ।
**Thank you very much for helping us with this research. Your comments will help to ensure that the Nepali version of the Subjective Happiness Scale has been properly translated and can be used internationally and in clinical setting.*

==

Signature of Interviewer: ______________________________
**Signature of Interviewer*

15. Interviewer Comments (please indicate whether or not the participant required assistance filling out the questionnaire, and also list any comments you may have):
**Interviewer Comments (please indicate whether or not the participant required assistance filling out the questionnaire, and also list any comments you may have):*

(List your observations as interviewer here. Include body language of participants, and if they genuinely understood items or they were just trying please the interviewer or get trying to complete this task done sooner.)

____

____

____

____

____

____

____

____

____

____

____

____

Supplementary Material - S2. Results of normal distribution for study variables.

Supplementary Material - S2a. Table for scores of skewness and kurtosis for study variables used in construct validity

Variables	N	Minimum	Maximum	Mean	SD	Skewness		Kurtosis	
						Statistic	SE	Statistic	SE
SHS	179	4	21	15.96	3.18	-0.54	0.18	0.28	0.36
PSFS	151	2	30	15.32	5.63	0.32	0.20	-0.13	0.39
Sleep Disturbance	180	8	40	19.78	6.66	0.23	0.18	-0.26	0.36
Depression	180	8	38	16.06	6.36	0.59	0.18	-0.15	0.36
Pain Intensity	180	3	15	9.39	2.02	-0.09	0.18	0.09	0.36
CDRISC	180	1	8	5.70	1.25	-0.68	0.18	1.19	0.36
PSEQ	180	3	60	47.16	11.15	**-1.25**	0.18	1.76	0.36
Quality of Life	180	4	10	6.83	1.04	0.37	0.18	-0.18	0.36
Pain Interference	180	6	29	15.01	5.16	0.18	0.18	-0.41	0.36
PCS	180	0	52	18.24	10.3921	0.39	0.181	-0.11	0.36

Abbreviations: SHS, Subjective Happiness Scale; PSFS, Patient-Specific Functional Scale; CDRISC, Connor-Davidson Resilience Scale; PSEQ, Pain Self-Efficacy Scale; PCS, Pain Catastrophizing Scale; SD, Standard Deviation; SE, Standard Error.

Note: For construct validity using hypotheses testing data for all the outcome measures were normally distributed based on visual inspection of histograms and both kurtosis (normally distributed if the value ranged from -1 to 1) and skewness (normally distributed if the value ranged from -2 to 2) statistics. All of the skewness and kurtosis values were within the normal range except for Pain Self-Efficacy Questionnaire (bold text).

Supplementary Material - S2b. Normal distribution curves for all study variables for Construct Validity.

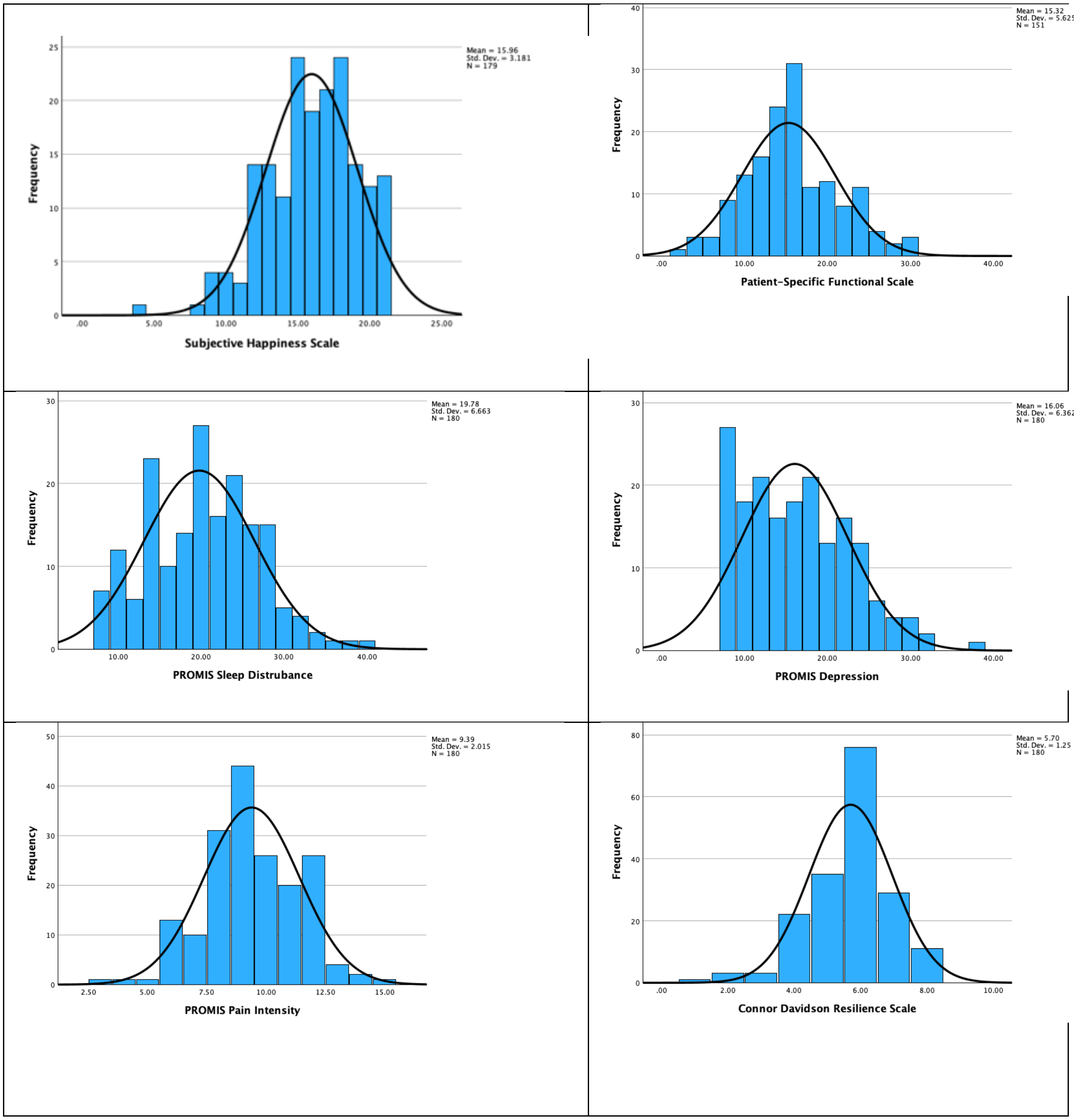

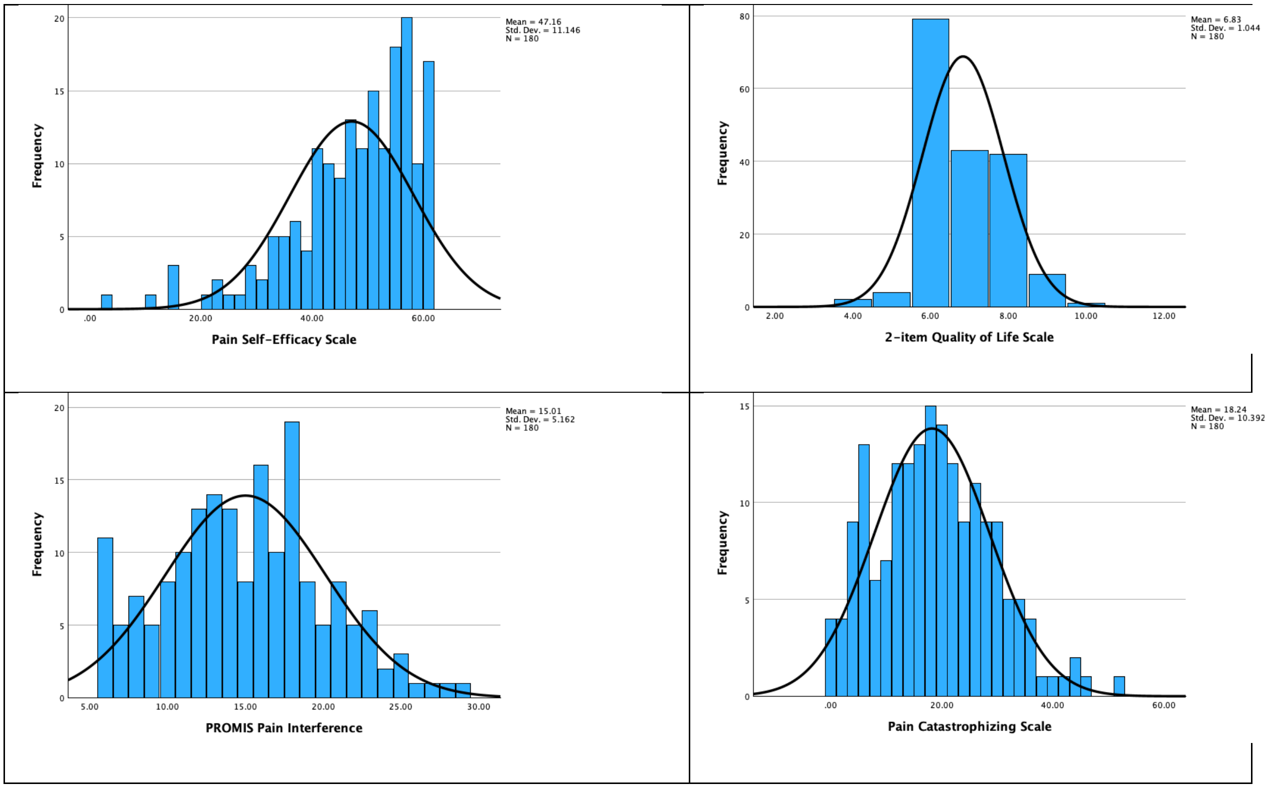

Mean = 47.16
Std. Dev. = 11.146
N = 180
Frequency
Pain Self-Efficacy Scale
Mean = 6.83
Std. Dev. = 1.044
N = 180
Frequency
2-item Quality of Life Scale
Mean = 15.01
Std. Dev. = 5.162
N = 180
Frequency
PROMIS Pain Interference
Mean = 18.24
Std. Dev. = 10.392
N = 180
Frequency
Pain Catastrophizing Scale

Supplementary material - S3. Key issues in the Nepali translation of SHS and their solutions

Problem site	Key translation issues and solution	Final translation
Instruction<br>For each of the following statements and/or questions, please circle the point on the scale that you feel is most appropriate in describing you.	The instruction was relatively simple, and therefore, easy to translate.<br>However, during the pre-testing interview, 4 out of 14 participants could not understand the Nepali translation of the word "describing." Therefore, another Nepali translation of the Nepali word "describing" was used.	For each of the following statements and/or questions, please circle the point on the scale that you feel is most appropriate in describing you.<br><br>तलको प्रत्येक वाक्यहरुमा वा प्रश्नहरुमा तपाईलाई बयान गर्ने अङ्क पहिचान गरी त्यसमा गोलो चिन्ह लगाउनुहोस् ।
Item 3<br>Some people are generally very happy. They enjoy life regardless of what is going on, getting the most out of everything. To what extent does this characterization describe you?<br>1 = not at all<br>7 = a great deal	It was difficult to find the right Nepali words for "getting the most out of everything." Therefore after a comment from MPJ, the phrase was translated as "getting satisfaction out of everything they do."<br>During pre-testing, four middle-aged participants, especially from the Newari ethnicity (2 out of 3 from the Newari community, one from Chhetri, and one from Dalit), had difficulty understanding the Nepali-translated words "characterization" and "describe." To overcome this challenge, researchers came up with a new question, including simpler words.	Some people are generally very happy. They enjoy life regardless of what is going on, getting satisfaction out of everything they do. Is this someone like you?<br><br>कोही व्यक्तिहरु सामान्यतया धेरै खुसी हुन्छन् । जीवनमा उनीहरु जस्तोसुकै परिस्थितिमा पनि रमाउदै आफूले गरेको सबै कुराहरुबाट सन्तुष्टि लिन सक्छन् । के तपाई यस्तो व्यक्ति हुनुहुन्छ ?
Item 4<br>Some people are generally not very happy. Although they are not depressed, they never seem as happy as they might be. To what extent does this characterization describe you?<br>1 = not at all<br>7 = a great deal	Item 4 was long and reverse-coded. It was among the most difficult items for the expert committee to translate.<br>During pre-testing, 13 out of 14 participants failed to understand item 4.<br>The scale developer suggested removing this item from the scale.	Item 4 was removed.

Note: *Problematic translations are given in bold font on the issues site.*

Supplementary Material - S4: Exploratory Factor Analysis of 3-item SHS

	**Total**	**Hard copy**	**Online**
KMO	0.74	0.74	0.71
Chi-Square	261.6	210.25	56.92
Degree of freedom	3	3	3
Significance	<.001	<.001	<.001
**Factor loading**			
Item 1	0.89	0.92	0.84
Item 2	0.89	0.92	0.84
Item 3	0.88	0.89	0.87

Abbreviations: KMO, Kaiser–Meyer–Olkin; SHS, Subjective Happiness Scale.

Please note that item 4 of Nepali version of the Subjective Happiness Scale was removed during the cognitive debriefing interviewing, therefore, it was not subjected to factor analysis assessment.
